# Editorial Brevities

**Published:** 1888-12

**Authors:** 


### EDITORIAL BREVITIES.

THE advertisement of Parke, Davis & Co., on fourth cover page, is an interesting one and will repay perusal.

BRYCE'S VISITING LIST AND POCKET RECORD.—Good for any month or year. Edited and published by C. A. Bryce, M. D., Editor Southern Clinic author of Medical Advice to Young Men. Southern Clinic print, Richmond, Va. Price $1 00. 1888. Contains poisons and antidotes, examination of urine, metric system, incompatibles, rules for doses, new remedies, dose list, etc. This is a cheap, neat and very convenient pocket record for a physician.

WE invite special attention to the advertisement of Lehn & Fink, commencing in this number of our journal. There Papain or new vegatable pepsin is favorably mentioned by members of the profession, and so is their extract of malt and aromatic tinct. of iron.

INEBRIATE ASYLUM IN GEORGIA.—We learn that the committee appointed to report upon the subject of an Inebriate Asylum in Georgia, have reported favorably, and there is a strong probability that the Legislature of Georgia will take favorable action in the matter at its present session. We think the measure would meet with the approval of a majority of the people, and certainly of almost the entire medical profession; for it is now conceded by medical men everywhere, that drunkenness, in certain of its phases, is a disease demanding seclusion, freedom from temptation, strict hygienic regulation and skilled treatment for its cure.

MEDICAL NEWS VISITING LIST 1889. Lea Brothers & Co., Philadelphia. This is excellently and admirably arranged for memoranda of accounts, etc. Contains table for ascertaining day of confinement. Thermometric scales,mteric system, weights and measures, examination of urine, disinfectants, remedies in use, incompatibles, artificial respiration, poison antidotes, table of doses, therapeutic table, etc., etc.

LAXATIVE IN CHILDBED—INFORMATION WANTED.—A medical friend wants to know what is the best and safest laxative to open the bowels after delivery. How long should we wait before moving the bowels, and what purgative is most desirable? It is often a quesiion, after severe labor, whether or not a purgative on the 2d or 3d day, as usually prescribed, will not do more harm than good. The disturbance of the woman before she is sufficiently strong, and the danger of affecting the milk so as to injure the babe are factors that enter into the subject. We hope that our subscribers, a few of them at least, will respond to this important and practical inquiry. Let everyone who knows any formula or remedy especially adapted to the opening of the bowels in childbed, send it to us and we will place it in the RECORD for the benefit of the profession.

